# Neuromorphic on-chip recognition of saliva samples of COPD and healthy controls using memristive devices

**DOI:** 10.1038/s41598-020-76823-7

**Published:** 2020-11-12

**Authors:** Pouya Soltani Zarrin, Finn Zahari, Mamathamba K. Mahadevaiah, Eduardo Perez, Hermann Kohlstedt, Christian Wenger

**Affiliations:** 1 IHP–Leibniz-Institut Fuer Innovative Mikroelektronik, 15236 Frankfurt an der Oder, Germany; 2 Nanoelectronics, Faculty of Engineering, Kiel University, 24143 Kiel, Germany; 3 BTU Cottbus-Senftenberg, 01968 Cottbus, Germany

**Keywords:** Biomedical engineering, Computational platforms and environments, Computational science, Chronic obstructive pulmonary disease, Diagnosis

## Abstract

Chronic Obstructive Pulmonary Disease (COPD) is a life-threatening lung disease, affecting millions of people worldwide. Implementation of Machine Learning (ML) techniques is crucial for the effective management of COPD in home-care environments. However, shortcomings of cloud-based ML tools in terms of data safety and energy efficiency limit their integration with low-power medical devices. To address this, energy efficient neuromorphic platforms can be used for the hardware-based implementation of ML methods. Therefore, a memristive neuromorphic platform is presented in this paper for the on-chip recognition of saliva samples of COPD patients and healthy controls. Results of its performance evaluations showed that the digital neuromorphic chip is capable of recognizing unseen COPD samples with accuracy and sensitivity values of 89% and 86%, respectively. Integration of this technology into personalized healthcare devices will enable the better management of chronic diseases such as COPD.

## Introduction

Chronic Obstructive Pulmonary Disease (COPD) is an inflammatory lung disease, causing breathing difficulties in patients due to obstructed airflow in lungs^1^. COPD is one of the main leading causes of death worldwide with an annual mortality rate of three million people^2^. Apart from its economical burden for healthcare systems, COPD drastically impacts patients’ life quality by restricting their physical activities. The main cause of COPD in developed countries is smoking tobacco, while lung damages caused by air pollution or scarce genetic conditions can also lead to the disease^1^. The most common symptoms of COPD include chronic coughs, chest tightness, shortness of breath, and abnormal sputum production. Despite the lack of an effective treatment for COPD, an early-stage diagnosis plays a crucial role for the effective management of the disease^3^. However, majority of patients with objective COPD go undiagnosed until late stages in the course of their disease due to the absence of necessary Point-of-Care (PoC) technologies. As a result, development of personalized solutions for the COPD management has been significantly promoted by contemporary healthcare systems for providing patients with appropriate medical assistance in an outpatient clinic or a home-care environment^4^.

Among various possible methods for the early diagnosis of COPD in a PoC setup, regular screening of dielectric properties of patients’ saliva has shown to provide important information on the disease status^5–8^. However, information obtained on this one single parameter, dielectric properties of saliva, is not sufficient by itself for providing a comprehensive diagnostic solution^9,10^. In other words, the accurate diagnosis of the disease based on this approach is only possible by concurrent consideration of various personal–medical parameters related to patients. These parameters include demographic information of patients such age, gender, and the smoking background. To address this issue, Machine Learning (ML) tools have been applied on the rudimentary information of saliva properties together with demographic parameters to identify the diagnostic status of patients in a PoC environment^9,10^.

ML tools applied on the clinical data acquired from PoC devices enable the efficient management of chronic diseases such as COPD. The scope of ML tools goes far beyond classical statistical analyses performed in medicine, providing accurate and real-time predictions on the health status of patients or the progress of their diseases^11,12^. In addition, availability of numerous health-related data, thanks to advancements in wearable technologies and internet-of-things, have facilitated the better integration of ML with healthcare devices in PoC environments^13^. Therefore, constant and remote monitoring of patients for the management of chronic and degenerative conditions, monitoring their rehabilitation progress, and predicting critical–emergency health conditions have become a reality^14^. For example, tracking heart activities of elderly, monitoring blood glucose levels in diabetic patients, and observing the rehabilitation progress of Parkinson’s diseased patients are all among recent applications of ML in PoC^11,13–16^. Moreover, by taking advantage of ML tools, valuable information can be extracted from the vast amount of user data for identifying previously unknown disease trends or diagnostic links and providing comprehensive treatment plans and recommendations for healthcare specialists^14,17^.

Although the astonishing performance of ML for various studies, shortcomings of cloud-based techniques have limited their real-world applications in medicine^18^. These shortcomings include data safety concerns related to securing sensitive medical data in a single database, susceptible to malicious attacks or scandals. In addition, complexities associated with cloud communications such as robustness against interference is another hurdle, requiring precise design for the short-range (device to smartphone via bluetooth) and long-range (smartphone to backend using internet) communications for transferring data from medical devices to the backend^13^. Furthermore, technical aspects of the cloud-based ML such as wide bandwidth requirements and low latency plays vital role for medical applications. For example, pre-processing and data curation—compression of the acquired data, prior to their extraction from a device towards the backend, is remarkably challenging for providing real-time information to users with the least possible delay. Last but not least, cloud-based techniques require immense energy consumption and enormous computational power, restricting their application for low-power PoC devices^9,19^.

Abovementioned shortcomings of the cloud-based ML in healthcare can possibly be addressed using neuromorphic platforms at the edge^19,20^. A neuromorphic platform offers a hardware-based imitation of neural networks by using actual electrical components as neurons and synapses^21^. Real-time analysis of data in a less time consuming manner with a smaller time delay is far more practical by bringing data post-processing from the backend onto a neuromorphic chip. Furthermore, securing sensitive medical data on a single chip, without cloud communications or backend storage, complies better with patient privacy regulations. In addition, since neuromorphic platforms process data near their source, they are relatively better immune against false operations and offer a large fault tolerance for medical applications. In other words, robustness of these technologies is vital for near-a-patient applications, where accessing a sufficient internet coverage is unfeasible. Moreover, energy-efficient hardware-based neuromorphic systems require less computational power, making them an adequate technology for edge-computing required in PoC medical devices^22–26^.

Considering remarkable advantages of neuromorphic chips, they have been recently investigated for various ML applications including in medicine. As a an example, Cai et al. have introduced a memristor-based neuromorphic computing chip for the breast cancer data classification^20^. The developed chip demonstrated high accuracy of 94.6% for the classification of benign and malignant samples within the breast cancer screening dataset. The computationally-efficient technology has enabled the real-time processing of data with high speed and low energy consumption. Similarly, Choi et al. have proposed a memristor-based neuromorphic crossbar array for the online clustering of breast cancer data in an unsupervised fashion^27^. In this work, principal component analysis algorithm was implemented on the chip for effectively classifying sensory data with 97.6% accuracy. Online learning was successfully achieved in the developed memristor network, demonstrating the practicality of using neuromorphics for performing complex ML algorithms required for data-intensive tasks such as medical pattern recognition. In another study, a spiking neural network was implemented on a neuromorphic chip for the real-time discrimination of electromyography signals for the hand gesture classification^28^. The proposed low-power technique provided an accuracy of 84% for the recognition of various gestures, making it a suitable technology for remote rehabilitation and diagnostic setups required for patients with Parkinson’s disease. Park et al. have reported the application of memristive neuromorphic synapses as a Hardware-based Neural Network (HNN) for the Electroencephalography (EEG) signal recognition^29^. Human thought patterns related to three different vowels were recorded using EEGs, while a subject imagined speaking them. Subsequently, the proposed memristive HNN system was applied for learning and recognizing patterns of the acquired EEG signals. The developed device provided high accuracy for extracting features through recorded EEG signals during speech imagination experiments. Apart from the mentioned biomedical applications, neuromorphic chips have been extensively used for imaging scenarios including digit recognition^30–35^. As reported by Wenger et al., learning and recognition of the MNIST dataset digits have been experimentally demonstrated by taking advantage of the inherent stochasticity of Complementary Metal Oxide Semiconductor (CMOS)-integrated memristive devices^30,31^. A notable recognition rate of 89%, for a MNIST subset, was achieved in this work, demonstrating the potential of the proposed Resistive Random Access Memory (RRAM) technology for performing complex ML tasks.

Although neuromorphic systems offer an alternative platform for edge-computing required for the execution of ML algorithms on portable medical devices, their time-consuming training procedure for learning complex medical patterns is a significant drawback^36,37^. Moreover, deployment of pre-trained sophisticated deep learning networks onto neuromorphic chips, with rudimentary network structures, leads to lower precision, increased latency, and degraded energy efficiency and accuracy^19,38^. Therefore, development of a neuromorphic-compatible network topology is significantly important for pre-training a simulation-based Artificial Neural Network (ANN) prior to its deployment on a hardware-based neuromorphic platform. Backend training of a neuromorphic-compatible ANN, on the cloud, reduces the training time required for learning complex medical patterns; while, implementation of the pre-trained network on a neuromorphic platform enables the real-time recognition and classification of medical data on a low-power PoC device^36,37^.

In our previous study, a neuromorphic-compatible ANN was developed for the COPD pattern recognition using synthesized data^9^. However, the hardware implementation of the model on a neuromorphic platform and its in-vitro performance evaluation using real clinical data were missing. Therefore, the objective of this work was to train our previously developed ANN simulation for the classification of saliva samples of COPD patients and Healthy Controls (HC) using real clinical data and to implement the trained ANN on IHP’s memristive hardware platform for on-chip recognition. The combination of the simulation-based training and hardware-based recognition facilitates the better integration of neuromorphics with PoC medical devices, required for the management of chronic diseases such as COPD. Moreover, neuromorphic-equipped healthcare technologies provide the best platform for patients to take advantage of ML-based medicine, while having control over their medical data and privacy.

## Methods

### Data preparation

**Figure 1:**
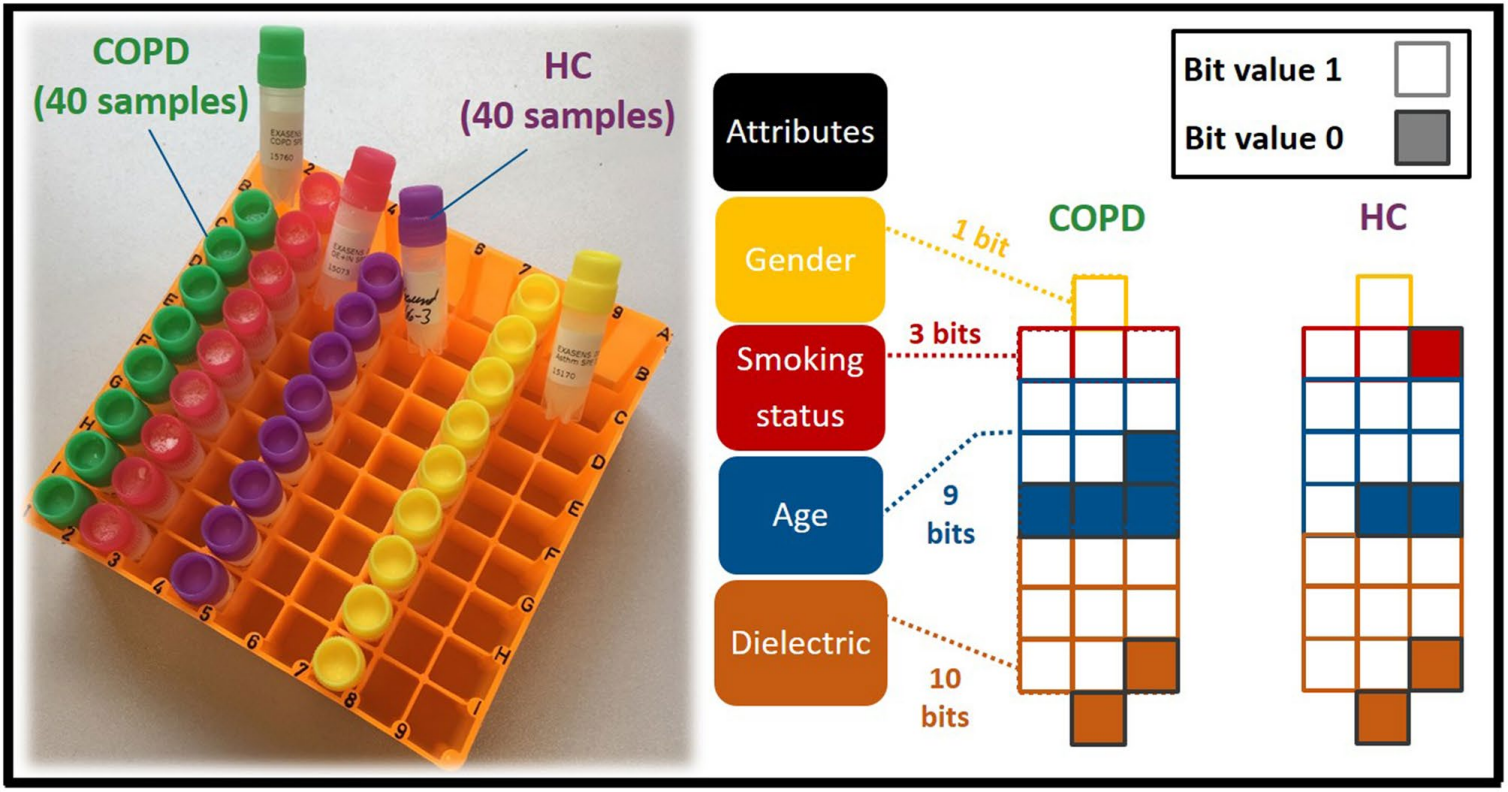
Conversion of analog attributes (gender, smoking status, age, and dielectric properties) into 23 binary bits.

The open access Exasens dataset, available at the UCI machine learning repository (https://archive.ics.uci.edu/ml/datasets/Exasens), was used in this study for training and evaluating the developed model for the classification and recognition of saliva samples of COPD patients and HC^10^. This novel dataset contains information on hundred saliva samples collected from four groups of respiratory patients including: COPD (40 samples), HC (40 samples), asthma (10 samples), and respiratory infected subjects without COPD or asthma (10 samples). Attributes of the dataset, used for the classification of subjects, include demographic information of patients (age, gender, and smoking status) as well as dielectric properties (Minimum value for the real part of permittivity) of the characterized saliva samples for every class. For computational purposes, non-quantitative attributes—diagnosis, gender, and smoking status—were converted into numerical values using following labels: diagnosis (COPD (1)–HC (0)), gender (male (1)–female (0)), smoking status (smoker (3)–ex-smoker (2)–non-smoker (1)). Subsequently, analog values of these four attributes were thresholded and converted into 23 binary bits (Gender (1), smoking status (3), age (9), dielectric permittivity (10)), as shown in Fig. 1. Binarization of attributes of this small-sized dataset has shown to reduce overfitting and noise, and to improve the performance of ML tools for the classification of COPD and HC samples^10,39^. In addition, considering the small size of the investigated dataset, 80 samples for two classes of COPD and HC, 5-fold cross-validation method was implemented for the evaluation of the ANN model, thus preventing overfitting and providing reliable and generalizable results. Therefore, for every cross-validation fold, the dataset was split into different test–train subsets with the ratio of 20–80%, respectively. The test-fraction, with unseen data points during model training, was considered as an external validation dataset for the evaluation of models. Data preparations and ML implementations were performed on the JupyterLab environment using Keras 2.2.5 and Scikit-learn 0.22 libraries of Python^40^.

### Artificial neural network

**Figure 2:**
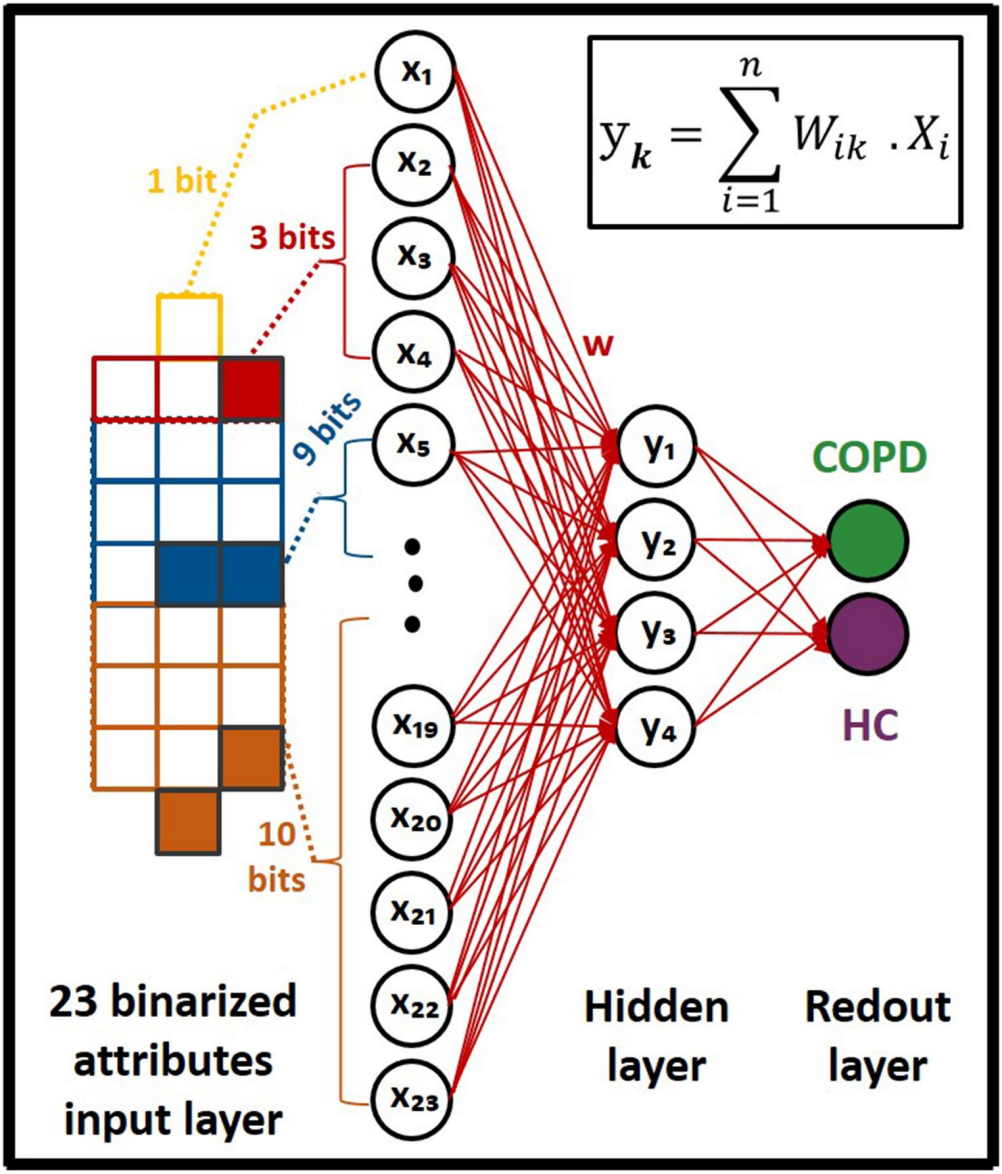
ANN simulation topology, with one hidden-layer, for the classification of saliva samples of COPD patients and HC.

**Figure 3:**
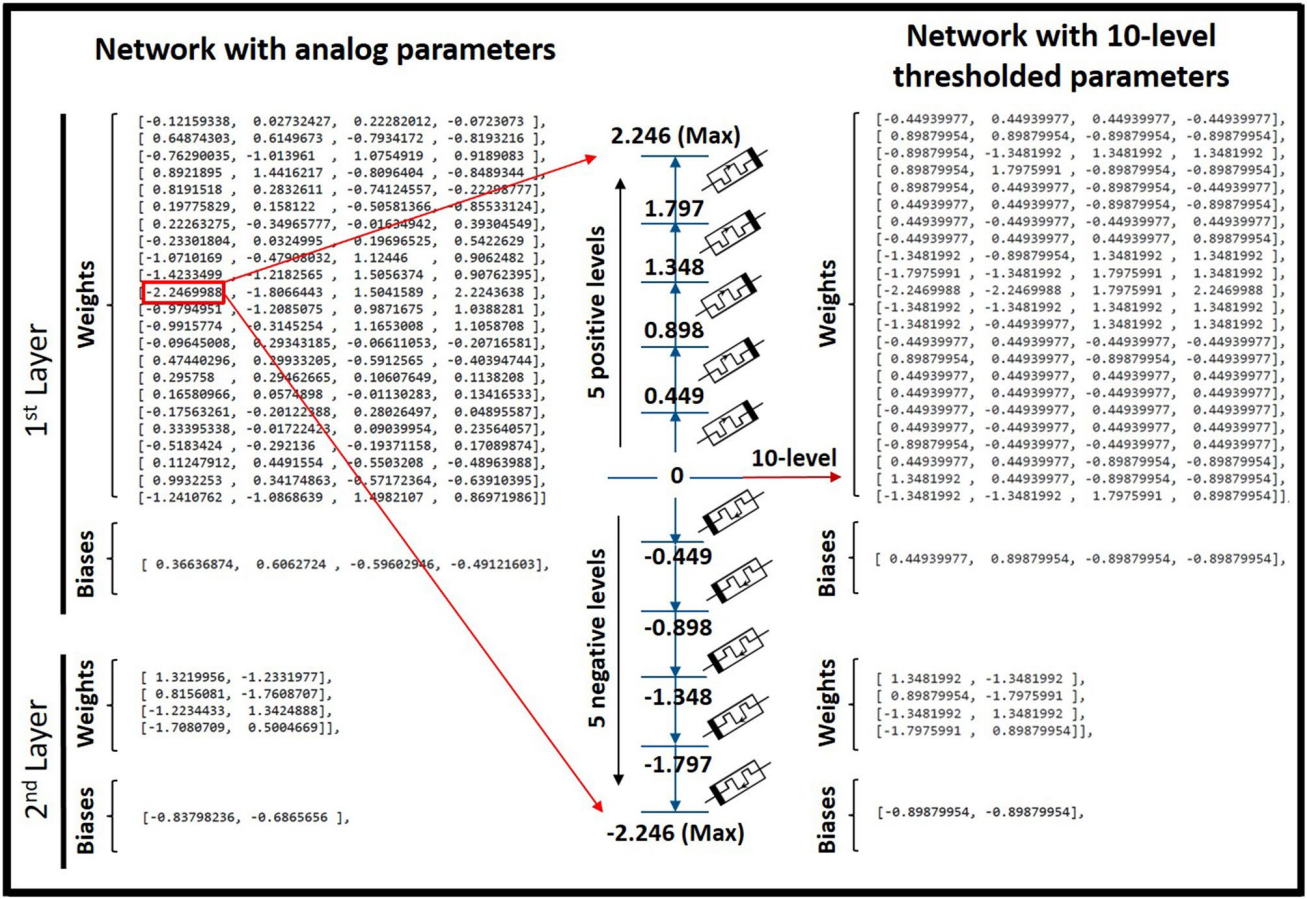
Multilevel thresholding of network analog parameters into 10 levels for complying hardware implementation requirements of the digital neuromorphic chip.

After data preparations, a dense ANN with one hidden-layer and one read-out layer was developed for the classification of COPD and HC samples, as shown in Fig. 2. The input layer of the network consisted of 23 neurons, considering binarized attributes of the dataset. To replicate the intrinsic structure of the intended neuromorphic platform, a hidden layer with 4 neurons and a sigmoid activation function was modeled. The read-out layer, with a sigmoid activation function, consisted of two neurons for two possible classes of COPD and HC. A dropout with 20% probability was applied to the hidden-layer for the overfitting prevention. Adam optimization algorithm, with 0.0001 learning rate, and a cross entropy error function were used for training network in the backend using the Google Colab GPU platform^41^. The developed ANN model was trained for 3000 epochs with a batch size of 10, using the train-subset of every cross-validation fold. Network parameters including weights and biases were computed and optimized during the training phase and their final analog values were recorded for every fold. Considering the fact that the intended neuromorphic chip consists of digital memristive devices, multilevel thresholding of network analog parameters into 10 levels was necessary for the deployment of the trained ANN onto the hardware platform. Therefore, calculated weights and biases of the trained ANN were thresholded into 10 levels to comply hardware requirements, as shown in Fig. 3. For this purpose, the absolute maximum value among calculated parameters was identified and divided by five to determine the resolution of thresholding levels. As shown in Fig. 3, the calculated threshold was used with positive and negative signs for the 10-level segmentation of network parameters with positive and negative values, respectively. After calculating thresholding steps, network parameters with analog values were shifted up to the nearest threshold value (for positive levels and equivalent for negative levels), representing one digital device per level. It is noteworthy that positive and negative levels are interpreted as devices with different current directions for the hardware implementation. Finally, converted weights and biases of the network with 10-level resolution were recorded and extracted for the deployment on the memristive neuromorphic platform, as shown in Fig. 3. All metrics and models are available in details at https://github.com/Pouya-SZ/Bioneuromorphics.

### Hardware implementation

**Figure 4:**
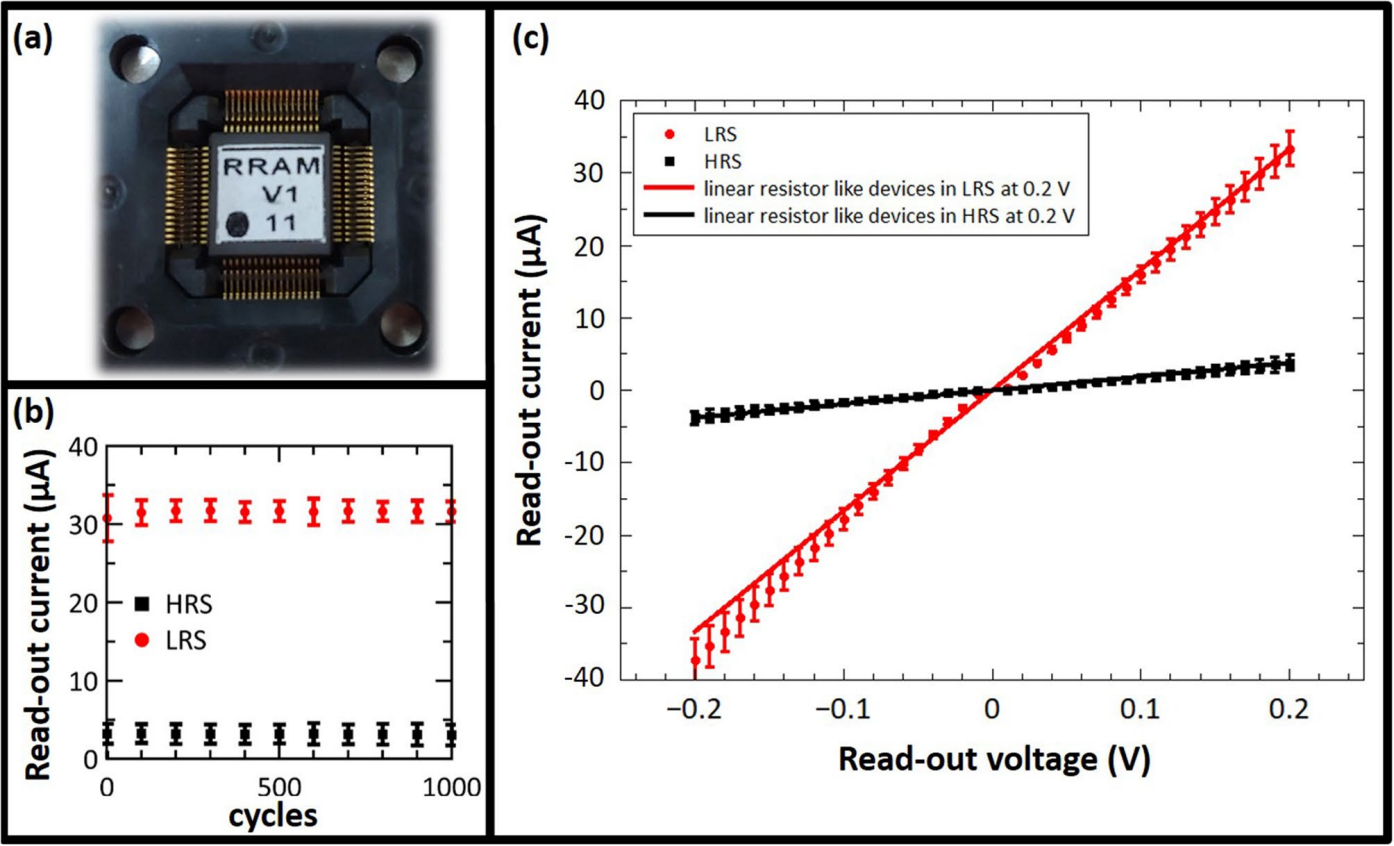
(**a**) 4-kbit CMOS-integrated RRAM array of IHP mounted on a PCB as synaptic weights in mixed-signal neuromorphic circuit; (**b**) Mean values and standard deviations of read-out currents of 128 devices integrated in a 4-kbit chip read-out at 0.2 V for 1000 switching cycles; (**c**) Read-out currents dependent on the read-out voltage amplitude and polarity. Red dots and black squares denoting mean values of 128 devices in LRS and HRS, respectively, while error bars are depicting standard deviations. The solid lines are showing linear resistors with resistance values similar to devices measured at +0.2 V in LRS (red) and HRS (black).

The hardware implementation of the developed model was performed on amorphous $\hbox {HfO}_2$ memristors which are CMOS-integrated 4-kbit RRAM arrays fabricated using the 250 nm CMOS technology at IHP^42–44^. The integration in CMOS technology is an important step towards fully integrated neuromorphic circuits. The array consists of $64\times 64$ memristive cells in a 1-Transitor-1-Resistor (1T-1R) configuration. The packaged chip is shown in Fig. 4a. Devices can be switched between two distinct states, i.e. low resistance state (LRS) and high resistance state (HRS), by the formation and dissolution of a conductive filament consisting of oxygen vacancies. Nominal read-out currents are $30\,\mu \hbox {A}$ and $5\,\mu \hbox {A}$ at 0.2 V for LRS and HRS, respectively. The evolution of mean read-out currents of 128 devices is shown in Fig. 4b. Here, two distinct states are clearly present for 1000 switching cycles. Mean read-out currents of $30.8\,\mu \hbox {A}$ and $3.2\,\mu \hbox {A}$ at 0.2 V for LRS and HRS, respectively, are changing marginally to $31.6\, \mu \hbox {A}$ and $3.0\,\mu \hbox {A}$. Mean read-out currents for different read-out voltages $V_{read} \le 0.2 \,\hbox {V}$ are shown in Fig. 4c for 128 devices being in LRS and HRS, respectively. The resistance does not scale linearly with the voltage^42^. Using a sufficient high voltage of 1.3 V or higher leads to reliable switching to LRS while using a sufficient low voltage of $-1.6$ V or lower leads to reliable switching to HRS. An even better control on the switching event can be achieved by using the Incremental Step Pulse with Verify Algorithm (ISPVA), which was used in this work^45^. It should be noted that applying voltage pulses with lower absolute value of the amplitude leads to stochastic switching between resistance states, which can be exploited for stochastic learning of analog data^30,31^. The stochasticity in amorphous devices is lower than in polycrystalline devices. This can most probably be attributed to a more homogenous defect distribution in the amorphous devices^43^, which is why these devices are used for the work described here. A thorough characterization of the devices in terms of switching voltages, endurance, yield and retention is given in^31,43^.

For the deployment of the thresholded model with 10 levels, a mixed-signal neuromorphic circuit with software-based neurons and hardware synapses was used similar to those shown in^30^. The RRAM chip was connected via a standard 64 pin integrated circuit socket to a Printed Circuit Board (PCB). Visual Basic was used to simulate neurons on a conventional computer and to control the experimental setup. Furthermore, an Arduino Mega 2560 microcontroller board was used to serve the address pins of the RRAM chip. Read-out and switching pulses were applied using an Agilent E5263A Source Measurement Unit (SMU).

**Figure 5:**
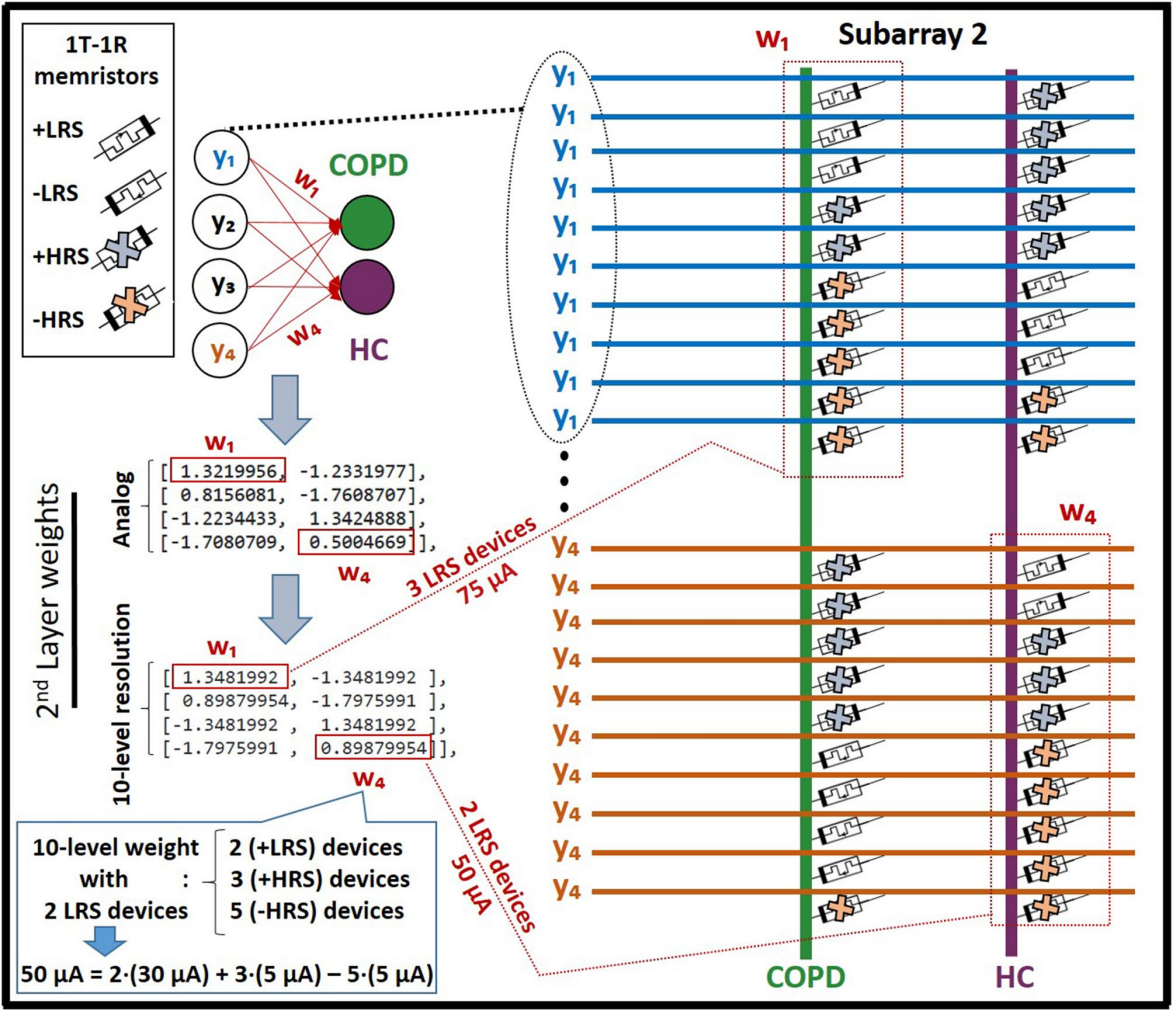
Assignment of memristive devices for the appropriate replication of simulation weights on the hardware, considering current values of $25 \,\mu \hbox {A}$, $50 \,\mu \hbox {A}$, $75 \,\mu \hbox {A}$, $100 \,\mu \hbox {A}$, and $125 \,\mu \hbox {A}$ for 1, 2, 3, 4, and 5 LRS levels (equivalent for negative levels), respectively.

Considering the topology of the developed ANN model (Fig. 2) with one hidden layer and one read-out layer as well as four and two neurons per layer, respectively, 106 parameters (i.e. synaptic weights and biases) were required for linking network layers. On the other hand, since every memristive device on the hardware represents one level of the thresholded parameters, 1060 memristive devices were required on the hardware for the implementation of the developed COPD recognition model with 10-level resolution. Resistance states of 1060 randomly chosen functional devices on a single chip were set to the HRS or LRS, respective to pre-trained weights. Every network parameter is represented by the combination of 10 devices so that the total value of the parameter is the sum of all 10 device currents read-out with voltages up to 0.2 V. Here, five devices are read-out with a positive voltage and five device are read-out with a negative voltage leading to positive and negative currents contributing to the total value of the network parameter. The minimum absolute value of one synaptic weight or bias is reached, when one device is in LRS and all other nine devices are in HRS leading to a nominal current of $1 \times 30 \,\mu \hbox {A} + 4 \times 5 \,\mu \hbox {A} - 5 \times 5 \,\mu \hbox {A} = 25 \,\mu \hbox {A}$ determined with 0.2 V. The maximum absolute value is achieved by switching all devices corresponding to the same read-out polarity to LRS while all others are in HRS. Thus, a nominal current of $5 \times 30 \,\mu \hbox {A} - 5 \times 5 \,\mu \hbox {A} = 125 \,\mu \hbox {A}$ is flowing. In between, equidistant discrete states can be achieved (shown in Fig. 5). Devices connecting input layer and hidden layer are read-out with $+0.2 \,\hbox {V}$ and $-0.2 \,\hbox {V}$, while devices connecting hidden layer and read-out layer are read-out with voltage amplitudes between –0.2 V and +0.2 V as it is explained below. This leads to a non-linear distortion of network parameters, because of the non-ohmic conduction mechanism depicted in Fig. 4c.

**Figure 6:**
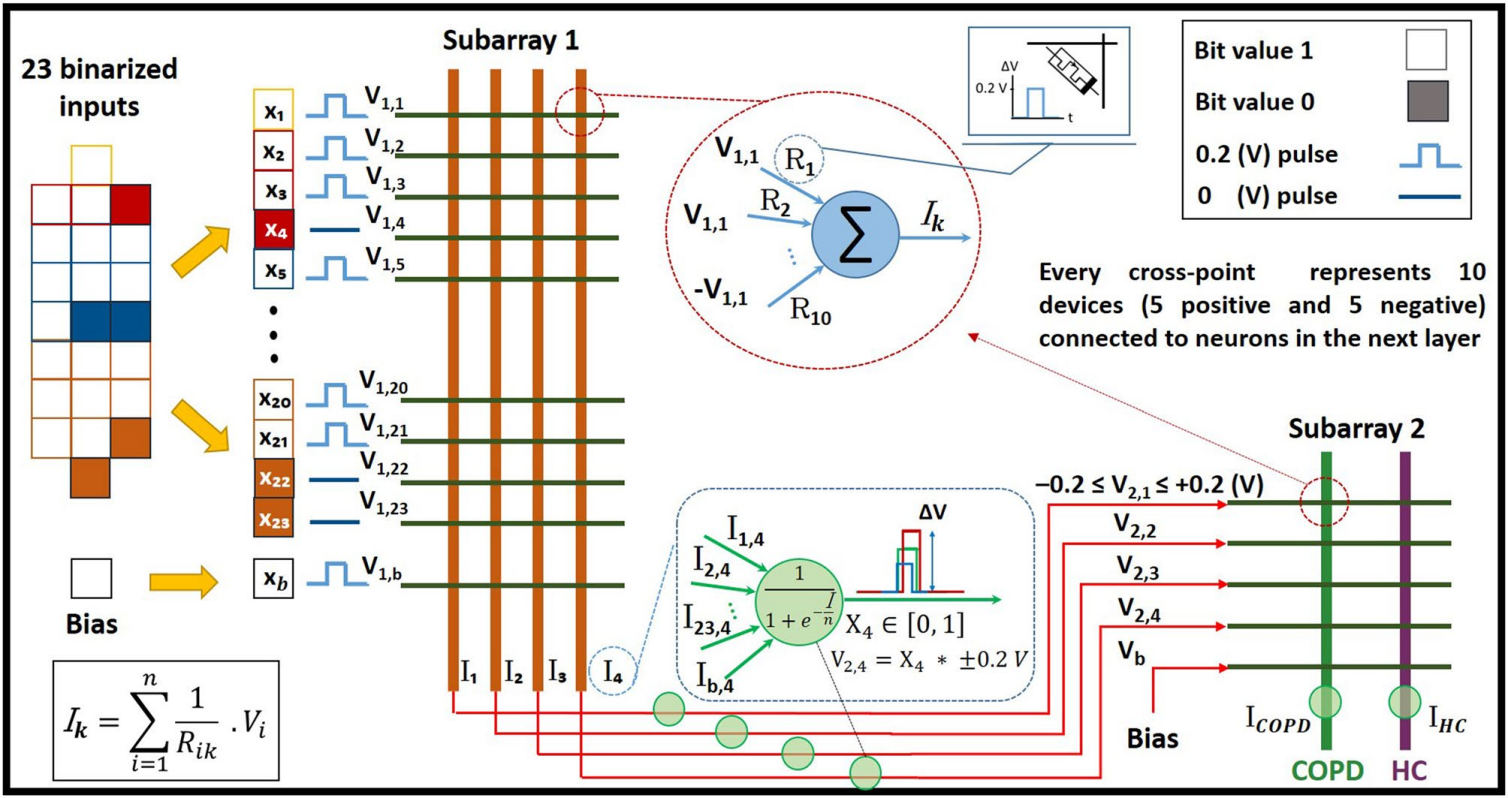
Application of 23 input bits of the test-subset data into simulated neurons for the recognition of COPD and HC samples. Input bits with a value 1 were applied to the network by voltage pulses of $\pm 0.2 \,\hbox {V}$ (i.e. $+0.2 \,\hbox {V}$ or $-0.2 \,\hbox {V}$ for devices assigned to a positive or negative contribution, respectively), while no voltage was applied for a 0 value input bit. Output neurons of every subarray are perceptrons with a sigmoidal activation function, which receive current values as the sum of all input voltages weighted with the resistivity values of individual memristive cells. These input currents are normalized by the factor *n* for mapping to a reasonable range for the activation of perceptrons. The output of the first perceptron layer is mapped to voltage pulses with variable amplitudes.

After the successful implementation of pre-trained weights on the hardware, the test-subset of data was used to evaluate the performance of the neuromorphic model for the recognition of COPD and HC samples. For the recognition of COPD samples with the mixed-signal approach, 23 input bits of the test-subset data were applied to simulated neurons on the input layer, as shown in Fig. 6. Input bits with a value 1 were applied to the network by voltage pulses of $\pm 0.2 \,\hbox {V}$ (i.e. $+0.2 \,\hbox {V}$ or $-0.2 \,\hbox {V}$ for devices assigned to a positive or negative contribution, respectively, as explained above), while no voltage was applied for a 0 value input bit. As shown in Fig. 6, output neurons of every subarray are perceptrons with a sigmoidal activation function, which receive the sum of current values passing through devices connected together with a specific bias value. The read-out of device currents is done serial and they are summed up in software. A parallel read-out would require an application specific chip design. Nevertheless, a proof-of-principle for using devices in a hardware neuromorphic circuit can be given using serial read-out. These current values are normalized by the factor *n* to the maximum value of the pre-trained analog network to guarantee the sigmoid function is activated with a reasonable range of values. Thus, the maximum current of $125 \,\mu \hbox {A}$ corresponds to the maximum pre-trained analog value. An activated perceptron *i* of the second layer is generating an analog output signals $\textit{X}_i$ within the interval of [0, 1]. These are applied to devices connecting layer 2 and 3 as voltage pulses with amplitudes $\textit{X}_i\cdot \pm 0.2 \,\hbox {V}$ with a precision of 10 mV. Output values of the third layer (read-out layer) perceptrons are denoting whether a test sample belongs to COPD or HC categories. This realization is in agreement with the theory of neural networks that the weighted sum of inputs determine the value of a perceptron neuron in the subsequent layer, as demonstrated in Fig. 2. Therefore, applying test-subsets of COPD and HC with different input patterns generated two different current values activating the read-out layer perceptrons of the neuromorphic network leading to the hardware-based recognition of these two classes.

### Performance assessment

**Figure 7:**
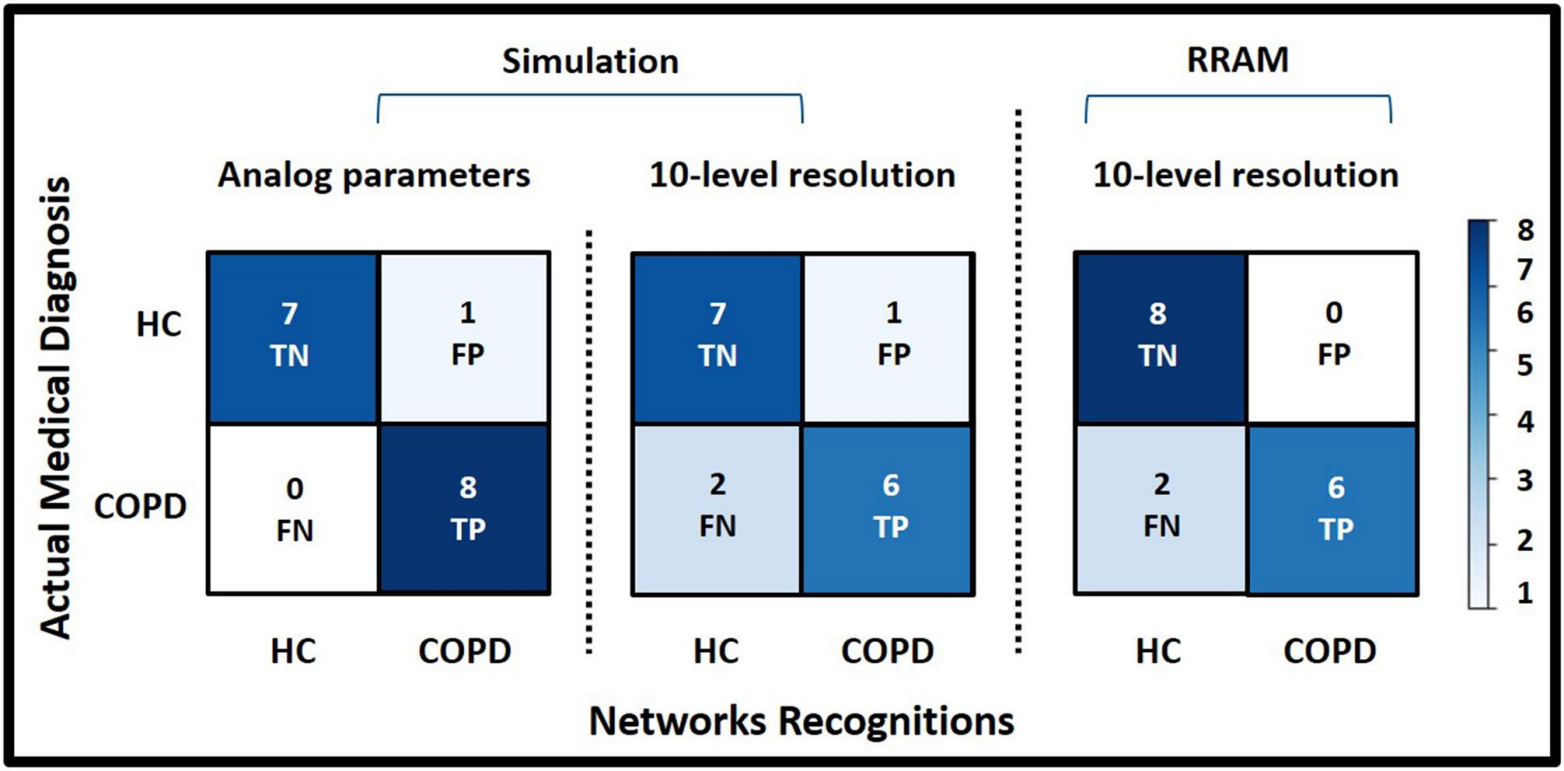
Confusion matrices for a single cross-validation fold (fold 5) for the recognition of COPD and HC samples, demonstrating the calculated sensitivity, precision, and specificity measures.

While the train-subset of every cross-validation fold (64 data points out of overall 80) was used for training the ANN and computing the 10-level model topology, the test-subset, with 16 data points, was considered as an external validation dataset for the performance evaluation of models for the recognition of saliva samples of COPD patients and HC. Tables 1, 2, and 3 report the 5-fold cross-validation performance of following models, respectively: ANN with analog parameters, ANN model with 10-level resolution topology, and the HNN with 10-level resolution deployed on the memristive neurmorphic chip. The performance measures reported in these tables include accuracy, sensitivity, specificity, and precision for every cross-validation fold as well as the average of all five folds. The reported accuracy measure in these tables indicates the performance of a model for correctly recognizing unseen test data, which was calculated as the percentage of true positives (correctly identified COPD) plus true negatives (correctly identified HC) out of all assessments. The sensitivity of a model was calculated as the proportion of True Positives (TP) out of all diseased cases; while the specificity value shows the number of True Negatives (TN) over number of TNs and False Positives (FP). Precision criterion shows the ratio of true positives over true plus false positives (incorrectly identified COPD). It should be noted that the hardware realization experiments were repeated five times for every single cross-validation fold to investigate the repeatability of measurements considering undesired effects of device-to-device variability and failed switching events. Therefore, results reported in Table 3 represent the average of five repetition for every single cross-validation fold. In addition, Fig. 7 demonstrates confusion matrices for a single cross-validation fold (fold-5).

## Results

As reported in Table 1, the ANN simulation with analog parameters provided a high accuracy of 90% for the recognition of unseen saliva samples of COPD patients and HC. In addition, sensitivity, specificity, and precision values of 92.5%, 87.5%, and 89.3%, respectively, have been reported for its 5-fold cross-validation performance, making it a reliable model for the in-vitro diagnosis of COPD. On the other hand, Table 2 presents the performance assessment of the ANN simulation with the 10-level resolution topology. Although a slight performance degradation compared to its original analog structure, the ANN model with 10-level resolution provided acceptable accuracy, sensitivity, specificity, and precision values of 87.5%, 85%, 90%, and 90.2%, respectively, for the recognition of unseen samples within the test-subset. These results are along with the fact that the multilevel thresholding of a network’s parameters impairs its recognition performance with respect to its resolution. In a similar manner, deployment of the model with 10-level resolution on the memristive neuromorphic platform has led to an on-chip recognition accuracy of 89%, indicating the reliability of the approach for the management of COPD in real-world applications (Table 3). In addition, high sensitivity, specificity, and precision values of 86%, 92%, and 92% have been reported for the on-chip recognition of 16 unseen test samples using the RRAM neuromorphic platform, making it a suitable technology for the implementation of ML techniques on low-power PoC medical devices. In particular, the network could reliably cope with the device-to-device variability of RRAM devices. Mean values of read-out currents were 3.9 ($\pm 1.0$) $\mu \hbox {A}$ and 35.5 ($\pm 3.7$) $\mu \hbox {A}$ for HRS and LRS devices, respectively. Additional to the device-to-device variability, failed switching events led to devices in the wrong resistance state. On average 2.68 of devices (i.e. 0.25%) were in the wrong state in each experimental run. Furthermore, the non-linear response of devices connecting layer 2 and 3 to voltage pulses with amplitudes between $-0.2 \,\hbox {V}$ and $+0.2 \,\hbox {V}$ (shown in Fig. 4c) did not strongly influence the accuracy. Even though all three mechanisms, i.e. device-to-device variability, failed switching events, and non-linear read-out may have affected the recognition performance of experiments, the overall average recognition rate was only slightly below the simulation with analog values. Nevertheless, in order to reduce the performance gap between the ANN simulation and its hardware-based replication on the chip, development of binary ANN models is necessary in the future^46,47^. Alternatively, analog neuromorphic platforms, capable of replication of analog parameters on-chip, can also be used to address this issue^48^.

**Table 1:** Performance of the ANN with analog parameters.

K-fold	Accuracy (%)	Sensitivity (%)	Specificity (%)	Precision (%)
Fold 1	87.5	100	75	80
Fold 2	93.75	87.5	100	100
Fold 3	81.25	87.5	75	77.78
Fold 4	93.75	87.5	100	100
Fold 5	93.75	100	87.5	88.89
Average	90	92.5	87.5	89.3

Figure 7 shows confusion matrices for the fifth cross-validation fold for the recognition of COPD and HC samples. The high accuracy, sensitivity, specificity, and precision for both simulation- and hardware-based ML models make them a promising tool for the recognition and management of COPD in PoC environments. Therefore, acquired results illustrate the practicality of using a memristive neuromorphic platform for the on-chip recognition and classification of saliva samples of COPD patients and HC using real clinical data, which was proposed as the objective of this work.

Results reported in Tables 1, 2, and 3 present similar trends for all five cross-validation folds, indicating the reliability of the model performance in terms of overfitting. However, similar to any ML study on a small-sized dataset, generalizability of presented results to a larger population of samples is the main limitation of this work, necessitating the extensive collection of data for the management of COPD. Nonetheless, to the best of our knowledge, there is no other comprehensive dataset available up to date, which can be used for training and evaluating the proposed neuromorphic-oriented ML models for COPD detection in this work. Therefore, we consider our study as a stepping stone to future studies in the field.

Neuromorphic platforms address the high energy consumption shortcoming of cloud-based ML techniques for edge-computing applications. The read-out of all synaptic weights throughout experiments consumed on average 614.2 ($\pm 60.3$) nJ per sample with a read-out pulse duration of $500 \,\mu \hbox {s}$ (i.e. the shortest pulse duration of the used Agilent E5263A SMU). This could be significantly reduced to 12.3 ($\pm 1.2$) nJ using $10\,\mu \hbox {s}$ pulses, which also allows a reliable read-out for utilized devices^44^. The energy efficiency of neuromorphic systems makes them an adequate technology for the integration of ML tools with low-power PoC medical devices. Hence, additional circuitry for a complete hardware realization (e.g. perceptrons) have to be implemented in low power electronics in the future.

## Discussion

It is noteworthy that the recognition accuracy of the neuromorphic HNN model improves with a greater number of thresholding levels, thus better replicating its original analog structure. However, a greater number of thresholding levels requires larger number of memristive devices on the hardware depending on the complexity of the original network and its number of parameters, thus restricting the resolution that can be chosen for the hardware deployment with respect to that specific neuromorphic hardware (e.g. 4096 devices for IHP’s RRAM chip). In addition, greater number of devices on the hardware consume more energy, while requiring a larger chip size and a longer time span for training and executing models depending on the internal design of the chip. Therefore, the trade-off between the accuracy of models and their on-chip efficacy in terms of power consumption and chip size for various thresholding resolutions need to be taken into account for determining the most optimum resolution.

**Table 2:** Performance of the ANN with 10-level resolution.

K-fold	Accuracy (%)	Sensitivity (%)	Specificity (%)	Precision (%)
Fold 1	87.5	87.5	87.5	87.5
Fold 2	93.75	87.5	100	100
Fold 3	81.25	87.5	75	77.78
Fold 4	93.75	87.5	100	100
Fold 5	81.25	75	87.5	85.7
Average	87.5	85	90	90.2

**Table 3:** Performance of the memristive neuromorphic chip.

K-fold	Accuracy (%)	Sensitivity (%)	Specificity (%)	Precision (%)
Fold 1	88	90	88	88
Fold 2	95	88	100	100
Fold 3	81	88	75	78
Fold 4	94	88	98	97
Fold 5	88	75	100	100
Average	89	86	92	92

As previously highlighted, for the sake of time efficacy, no learning or training was performed on the neuromorphic chip in this work. This is due to the fact that training a simulation-based model for many thousand iterations in the backend is far more practical. For instance, training the ANN model, in this study, for the classification of COPD and HC samples required an average time span of 250 seconds for 3000 learning epochs. Therefore, this work illustrates promising results for the practicality of using pre-trained neuromorphic chips in complex real-world applications, such as imaging, with time-consuming training requirements. Nevertheless, RRAM neuromorphic systems can also be used for on-chip learning and adaptation to new input patterns by developing network structures and acquiring algorithms^30,37^. Adaptability of these chips to an individual patient data is significantly important for applications such as the epileptic seizure prediction, where developing a generalizable ML model is not possible^38^. Hence, by taking advantage of neuromorphic-based ML techniques, developing personalized solutions for the management and diagnosis of chronic diseases such as COPD is feasible.

Notable results of this work imply the feasibility of using neuromorphic-based ML techniques for the enhancement of PoC healthcare solutions for the management of COPD. Energy-efficient neuromorphic systems, used in this work, are expected to revolutionize the ML-based medicine in the future by bringing data post-processing from the backend onto the chip, thus providing accurate and real-time predictions on the health status of patients. Furthermore, neuromorphic-equipped medical devices will better protect users’ sensitive medical data without cloud communication requirements. Moreover, implementation of novel meta learning algorithms, such as few-shot learning, on neuromorphic platforms will enable the rapid adaptation and real-time learning in these systems with a few data points and the least possible computation^39,49^. Example of such applications, where online learning and adaptation of a ML model is crucial, include autonomous driving, surgical robotics, personalized medicine, and precision diagnostic^39,49–52^.

In conclusion, this work investigated the concept of on-chip recognition of saliva samples of COPD patients and HC using a memristive neuromorphic platform. A hardware-friendly artificial neural network simulation was developed and trained in the backend for the classification of COPD and HC samples using real clinical data. Subsequently, analog parameters of the trained model were thresholded into 10 levels and were deployed on a memristive neuromorphic platform for on-chip recognition purposes. The neuromorphic chip with 10-level resolution provided a remarkable accuracy of 89% for the on-chip recognition of COPD and HC samples, offering an alternative approach to cloud-based ML methods required for the management of COPD in PoC environments. As the next step, a binary ANN model for the prediction of epileptic seizure will be developed and deployed on the introduced memristive neuromorphic system for the on-chip forecasting of epilepsy scenarios using low-power healthcare wearables.

## Data Availability

Data used in this work are available publicly at the UCI machine learning repository under the open access Exasens dataset https://archive.ics.uci.edu/ml/datasets/Exasens. All metrics and models of this work are accessible at https://github.com/Pouya-SZ/Bioneuromorphics.
